# A Combined NMR-Computational Study of the Interaction between Influenza Virus Hemagglutinin and Sialic Derivatives from Human and Avian Receptors on the Surface of Transfected Cells

**DOI:** 10.3390/ijms19051267

**Published:** 2018-04-24

**Authors:** Francesca Vasile, Maddalena Panigada, Antonio Siccardi, Donatella Potenza, Guido Tiana

**Affiliations:** 1 Department of Chemistry, University of Milano, Via Golgi 19, 20133 Milano, Italy; donatella.potenza@unimi.it; 2 Molecular Immunology Unit, San Raffaele Research Institute, via Olgettina 58, 20132 Milano, Italy; panigada.maddalena@hsr.it (M.P.); siccardi.antonio@hsr.it (A.S.); 3 Center for Complexity and Biosystems and Department of Physics, University of Milano and INFN, Via Celoria 16, 20133 Milano, Italy; guido.tiana@unimi.it

**Keywords:** STD-NMR spectroscopy, influenza virus Hemagglutinin, ligand–protein interaction, transfected cells, antiviral drug discovery.

## Abstract

The development of small-molecule inhibitors of influenza virus Hemagglutinin could be relevant to the opposition of the diffusion of new pandemic viruses. In this work, we made use of Nuclear Magnetic Resonance (NMR) spectroscopy to study the interaction between two derivatives of sialic acid, Neu5Ac-α-(2,6)-Gal-β-(1–4)-GlcNAc and Neu5Ac-α-(2,3)-Gal-β-(1–4)-GlcNAc, and hemagglutinin directly expressed on the surface of recombinant human cells. We analyzed the interaction of these trisaccharides with 293T cells transfected with the H5 and H1 variants of hemagglutinin, which thus retain their native trimeric conformation in such a realistic environment. By exploiting the magnetization transfer between the protein and the ligand, we obtained evidence of the binding event, and identified the epitope. We analyzed the conformational features of the glycans with an approach combining NMR spectroscopy and data-driven molecular dynamics simulations, thus obtaining useful information for an efficient drug design.

## 1. Introduction

Influenza viruses are important respiratory pathogens that have caused significant morbidity, mortality, and considerable economic losses in the recurrent yearly epidemics. Influenza viruses include several genera, such as A, B and C; however, only a few serotypes of the influenza A virus (IAV) have caused influenza pandemics in the last 100 years [1]. The influenza virions carry a lipid envelope embedding three surface proteins: hemagglutinin (HA), neuraminidase (NA), and the M2 proton channel protein. Based on the antigenicity of HA and NA, IAV can be further classified into different subtypes, including 16 HA (H1–H16) and 9 NA (N1–N9) subtypes. HA triggers the internalization into the host cell, upon binding with the sialic-acid-containing glycans on the host membrane. On the other hand, the role of NA is to release the newly formed virus particles from an infected cell.

Viral endocytosis initiates when HA binds to the sialic acid of the oligosaccharide moieties on the target membrane [2]. The glycan-recognition event controls the fusion of the membranes, and triggers the internalization and release of the viral genome into the cytoplasm. The receptors of HA display various glycosidic linkages between sialic acid (Neu5Ac: N-acetyl neuraminic acid), and the penultimate sugar of the chain. The most common of these are (α2–3) or (α2–6) linked to galactose (Gal) or N-acetyl galactosamine (GalNAc), (α2–6) linked to N-acetyl glucosamine (GlcNAc), and (α2–8) linked to another sialic acid residue. It has been shown that influenza viruses can recognize sialyl(α2–3)- or sialyl(α2–6)-linked disaccharide moieties, such as Neu5Ac(α2–3)Gal or Neu5Ac(α2–6)Gal, Neu5Ac(α2–3)GalNAc or Neu5Ac(α2–6)GalNAc, and Neu5Ac(α2–6)GlcNAc-containing oligosaccharides [3], but cannot bind to Neu5Ac(α2–8)-linked oligosaccharides [4].

**Figure 1:**
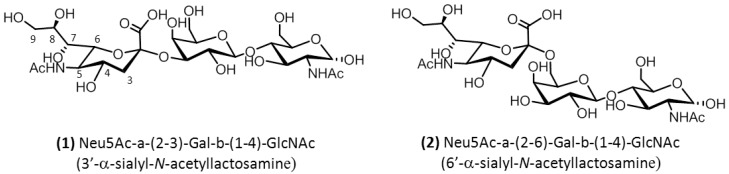
Structures of the α2–3 (**a**) and α2–6 (**b**) derivatives.

Thus far, the accepted paradigm for the determination of avian and human virus infection has been that human flu viruses preferentially bind to sialyl(α2–6)-linked disaccharides predominant in the upper human respiratory tracts, whereas avian flu viruses bind to sialyl(α2–3)-linked disaccharide moieties of the host receptor binding sites, which predominate in the avian enteric tract [3,5] (Figure 1). However, the paradigm of classifying viruses as only sialyl(α2–3) or (α2–6) linkage types does not seem to be sufficient to explain the infection and transmission of various strains of avian and mammalian influenza viruses. In fact, in the case of H5N1 and H7N9, as well as other mutated influenza viruses, the classification based on linkage specificity does not correlate to the susceptibility to infection, and the intra- and inter-species transmission of influenza viruses. For example, the highly pathogenic H5N1 viruses show strong binding properties to sialyl(α2–6) and weak binding properties to sialyl(α2–3), but both have shown a poor propensity for human infection and human-to-human transmission [6,7].

To date, several competitive inhibitors of NA (e.g., oseltamivir and zanamivir) have been developed; however, alternative anti-influenza drugs are urgently needed as a consequence of their ever-increasing tendency to induce drug resistance [8]. Lipids and polymers containing sialic acid have been studied as competitive entry blockers [9], but unfortunately they have been shown to suffer from a subtype-dependent antiviral activity, and a low barrier for resistance selection [10]. Taking advantage of these attempts, it became clear that there is a need to develop universal antagonists, able to bind the sialic binding site of HA with such a high affinity that single protein mutations could not jeopardize their potency.

In a previous paper, we developed [11] a new model system where HA was expressed on the surface of stably transfected human HEK-293T cells (Human Embryonic Kidney). The high-density expression of native HA was confirmed by monoclonal antibodies specific for their native trimeric conformation. By carrying out saturation-transfer difference (STD)-NMR experiments, we demonstrated that it is possible to quantify the interaction of a trisaccharide and two HAs expressed on HEK-293T cells. Our method overcame the problem of using recombinant solubilized viral membrane proteins, whose structure may not accurately reflect the native structure due to the absence of membrane components. On the other hand, it has advantages relative to virus-like particles (VLP), that could contain significant amounts of serum proteins, and could exhibit non-specific interactions, making the interpretation of NMR data concerning the ligand–target interactions particularly complicated.

In this paper, we analyzed two glycan-receptor-derived ligands, Neu5Ac-α-(2,3)-Gal-β-(1–4)-GlcNAc (compound **1**), and Neu5Ac-α-(2,6)-Gal-β-(1–4)-GlcNAc (compound **2**), displaying different glycosidic linkage between sialic acid (Neu5Ac: *N*-acetyl neuraminic acid) and the penultimate sugar of the chain (Galactose). Since the conformation of the glycan is important in order to determine the specificity of the receptor in the avian hemagglutinin subtype H5, we first analyzed the conformational features of these two trisaccharides in solution (Figure 1), and then studied their binding properties to avian H5 and human H1 subtypes. The overall goal of this study was to correlate the conformational properties of the ligands with their observed binding mode to HA.

When studying the conformation of the ligands by nuclear Overhauser effect (NOE) spectroscopy, we expected the two molecules to display a high degree of flexibility, and consequently, the interpretation of the NOE intensities in terms of molecular conformations was not straightforward. The standard way of obtaining spatial conformations from nuclear Overhauser effect speectroscopy (NOESY) experiments is to transform the experimental intensities into distance restraints, and to find the conformation that minimizes the violation of these restraints. The result of this procedure is an average structure, that, for flexible molecules, is poorly representative of the various ensembles of conformations among which the molecule fluctuates at equilibrium. Even when multiple conformations are obtained from the minimization of the restraints, these do not represent the actual thermal fluctuations at equilibrium.

In order to generate a conformational ensemble compatible with the experimental NOE intensities and with the laws of equilibrium thermodynamics, we made use of the computational strategy reported in Reference [12]. In a nutshell, it consists of making iterative molecular dynamics (MD) simulations starting from a standard force field, and correcting them to match the measured NOE intensities to a quantitative grade (cf. also Section 4 below). In this paper, we applied this strategy to glycans for the first time. This strategy, like other analogous approaches [13,14,15], is based on the principle of maximum entropy, that is, it finds the least biased model that reproduces the experimental data. In fact, it is possible to show [12] that a correction to the force field displaying the same functional form as the model that predicts the observables from the simulated conformations (the so-called “forward model”), minimizes the subjective bias by maximizing the associated Shannon entropy. However, at variance with other approaches based on the maximum entropy principle, the present algorithm has direct control of the molecular force field, thus allowing one to correct it easily. However, the drawback of this kind of approach is that the resulting force field is usually system-dependent, and thus is not portable.

The complexes of glycans in the presence of cells overexpressing H5 and H1 hemagglutinin were analyzed by STD-NMR [16], an ideal tool with which to map the epitope and describe the target–ligand interactions [7,17,18], and by transfer-NOE spectroscopy (tr-NOESY) [19,20], which provides information about the ligand conformation in the bound state. We applied STD-NMR and tr-NOESY techniques directly to suspensions of living cells, as we have already done in order to study the interactions between small ligands and membrane-bound proteins in other systems [21,22,23,24].

In this paper, we demonstrated that this procedure is a valid method for screening the interactions between small molecules and HAs.

## 2. Results

### 2.1. Conformational Analysis of the Ligands in Solution

The analysis of the conformational properties of compounds **1** and **2** was particularly difficult because of the high mobility of these small saccharides, suggested by the presence of few NOE cross peaks. We analyzed the two molecules in their free state using a Bruker 400 MHz instrument, and measured the intensities of cross peaks of three NOESY spectra acquired with 700 ms of mixing time (Figure S1 in Supplementary Materials). 

**Figure 2:**
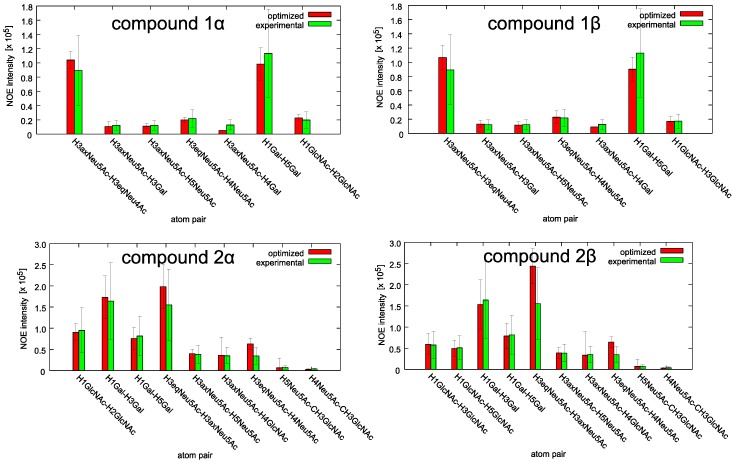
The experimental NOE intensities (green bars) are compared with those back-calculated from the molecular dynamics (MD) simulation after the optimization algorithm (red bars) for compound **1** (upper panels) and **2** (lower panels).

The conformational ensembles of the two compounds were obtained from the NOE intensities using the iterative computational approach described in Section 4 below. At the end of the calculation, the NOE intensities back-calculated from the model are within the error bars of the experimental data (Figure 2 and Figure S2 in the Supplementary Materials). The χ^2^ between model and experimental intensities is 0.6 for both anomers of compound **1**, and 0.7 and 1.1 for anomers α and β respectively of compound **2**.

**Figure 3:**
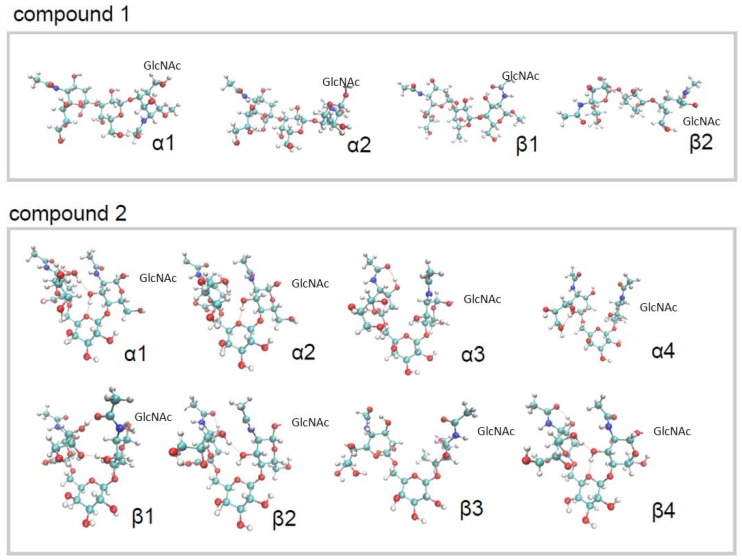
Conformational ensembles obtained for compounds **1** and **2**. Dashed red lines indicate hydrogen bonds.

**Figure 4:**
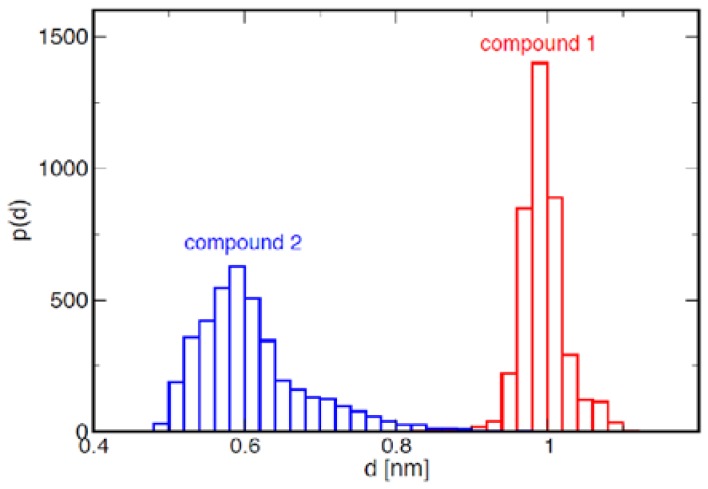
Distribution of distances *d* between the centers of mass of Neu5Ac and GlcNAc for compounds **1** and **2**, respectively.

The ensemble of conformations resulting from the calculation is quite heterogeneous for both anomers of the two molecules (Figure 3, see also Figure S3 in the Supplementary Materials). From the integration of the signals of the two anomers of GlcNAc on the 1D NMR spectra, we calculated the fraction of anomers α with respect to β, with the result of 65% for both compounds **1** and **2**. The conformational ensembles obtained for the two anomers of the same compound (Figure 3) were then merged according to the fractions reported above, resulting in a single equilibrium population for each compound. The distribution of distances between the centres of mass of Neu5Ac and GlcNAc is displayed in Figure 4. It is apparent that in compound **2,** the two termini of the molecule display large fluctuations from bent (*d* ≈ 0.6 nm) to extended (*d* ≈ 1 nm) conformations, but bent conformations are markedly more populated at equilibrium. By contrast, compound **1** displays fewer fluctuations, and populates only elongated conformations. No substantial difference was found in this behavior between anomers α and β (Figure S4 in the Supplementary Materials).

Selected conformations of the two compounds are displayed in Figure 3. Anomers of compound **1** display two major conformations, one more elongated due to the formation of hydrogen bonds between the oxygen of the acetyl group and OH in position 7 of Neu5Ac, and the oxygen in position 6 and OH3 of the GlcNAc, and the other slightly less elongated, lacking these hydrogen bonds. Compound **2** displays a more complex conformational ensemble, characterized by compact conformations stabilized by different combinations of the following hydrogen bonds: OH in position 3 of GlcNAc and the oxygen of the Galactose ring; the oxygen of the acetyl group and the OH4 of Neu5Ac; the oxygen in position 3 of GlcNAc and OH in position 7 of Neu5Ac; the oxygen in position 9 and OH7 of Neu5Ac. Other conformations, both bent and elongated, were identified for compound **2** lacking these hydrogen bonds (Figure 3).

Since the tr-NOESY of the ligands in the presence of cells is noisy due to the cells’ signals, we were not able to perform the same calculation for the bound form. However, the qualitative analysis of NOE cross peaks suggested that there is no difference between free and bound conformations.

### 2.2. NMR Interaction Studies

Making use of the STD-NMR technique, we analyzed the interaction of compounds **1** and **2** with avian H5 and human H1 proteins, expressed on the membrane of stably transfected 293T cells. Immunoprecipitation with antibodies CR6261 of membrane proteins, followed by Western blot analysis in non-reducing conditions, demonstrated that the surface proteins are correctly conformed as trimers. Furthermore, the HA molecules were shown to be able to bind sialic acid, as they agglutinate chicken red cells forming rosettes. Untransfected 293T cells were used as a negative control (no binding evidence was obtained in the experiment with control cell lines, Figure S6D). The use of the STD technique made it possible to show intermolecular binding, and to provide information about the protons involved in the epitope.

STD experiments were performed with cell suspensions in deuterated PBS buffer using approximately 10^7^ cells in the presence of ligand **1** or **2** (about 3 mM).

We repeated a hemagglutination test on cells in buffer, and confirmed that HAs maintain their conformation after 15 h (which is the NMR condition and experiments time).

**Figure 5:**
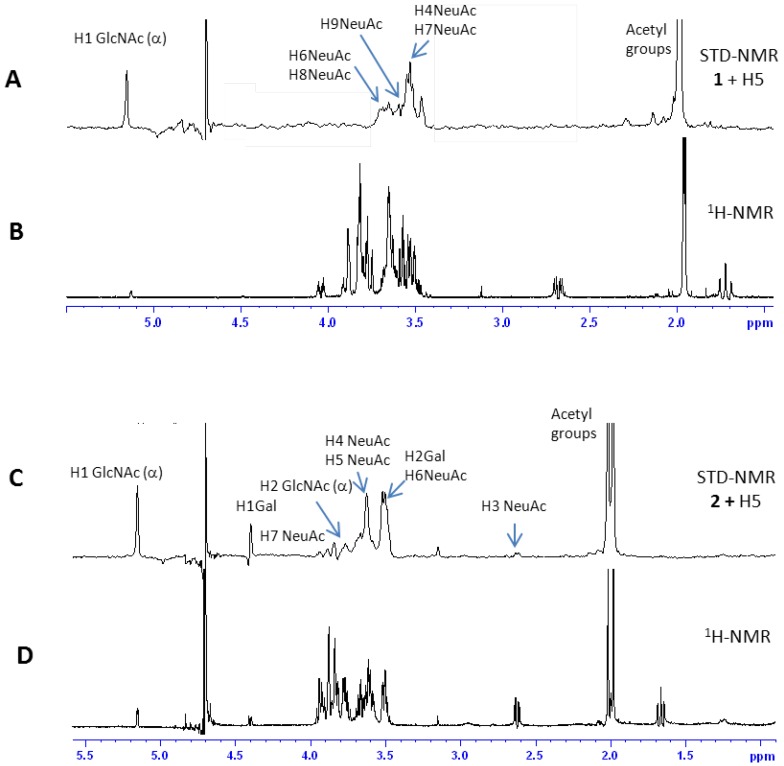
(**A**) STD spectrum of compound **1** in presence of cells expressing H5. (**B**) ^1^H-NMR spectrum of compound **1** in phosphate buffer. (**C**) STD spectrum of compound **2** in presence of cells expressing H5. (**D**) ^1^H-NMR spectrum of compound **2** in phosphate buffer.

STD spectra of both compounds in the presence of H5 are shown in Figure 5, while experiments in the presence of H1 are reported in Figures S5 and S6 of the Supplementary Materials. Blank experiments on ligands were carried out to assure the absence of direct irradiation of the ligands.

The relative intensities of the STD signals for each ligand can be quantified (I_STD_ = I_0_ − I_sat_, where I_0_ is intensity of the signal in the off-resonance experiments and I_sat_ is the intensity of the same signal in the on-resonance experiment), showing the proximity (high I_STD_) or distance (low I_STD_) of the proton to the receptor surface. The shorter the protein–ligand proton–proton distance (bound state), the stronger the intensity of the corresponding STD signal. Therefore, by calculating the absolute STD intensities, the so-called “group epitope mapping” is obtained (expressed as percentages).

#### 2.2.1 Interaction Studies in the Presence of H5

**Figure 6:**
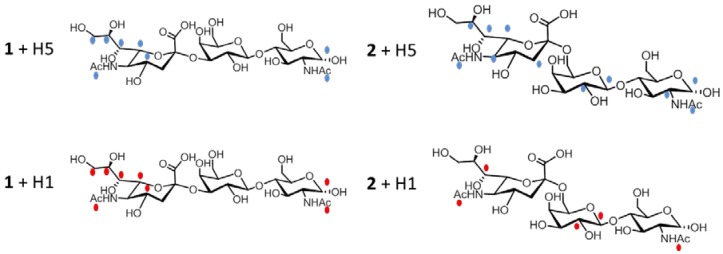
Protons of the epitope of compounds **1** and **2** in the presence of cells expressing HA are marked with blue dots for H5 and red dots for H1.

Figure 6 (upper panel) shows the epitope (the dots mark the hydrogen which gives such significant STD signals) for ligands **1** (left panel) and **2** (right panel) in the presence of H5. The high STD signal obtained in both cases for the anomeric proton of the reducing-end GlcNAc is most likely an artifact, due to the interaction with water.

In the case of Neu5Ac-α-(2,3)-Gal-β-(1–4)-GlcNAc (ligand **1**, upper left panel of Figure 6), data suggest that the entire terminal portion of the molecule is well inserted into the binding site, as a strong interaction is observed for the Neu5Ac residue. The epitope is determined by many strong STD signals, comprising H4 (the intensity of absolute STD is about 2%), the acetyl group (2.9%), H7 (about 2%), H9 (0.8%), and H6 and H8 (we cannot distinguish between these last two protons which are isochronous, and have an STD absolute intensity of about 0.4%). A STD signal was also observed for the acetyl group, and for the anomeric proton of GlcNAc at the reducing end of the trisaccharide.

Furthermore, in the case of Neu5Ac-α-(2,6)-Gal-β-(1–4)-GlcNAc (ligand **2**, upper right panel of Figure 6), the residue that is most involved in the interaction is the Neu5Ac moiety. In fact, several STD signals can be detected, such as the acetyl group, and the H3, H5, H6, H7, and H8 protons. The GlcNAc residue can interact with the protein, giving rise to the signals of the acetyl group, of the anomeric proton and of H2in the STD spectrum. The epitope is completed by the H1 and H2 protons of Gal residue. The large STD percentages suggest that a strong interaction involves all residues of the trisaccharide; three contacts of Nau5Ac produce a STD signal higher than 1%, and the acetyl groups of Neu5Ac and GlcNAc show strong signals as well. The H1 and H2 protons of Gal residue display a STD percentage higher than 1.5% (Figure S7 in Supplementary Materials).

These results suggest that the ligands **1** and **2**, derived from the HA natural receptor, can bind HA-H5 overexpressed on HEK cell membrane, on which the protein maintains its native/active conformation. As expected, the interaction is guided by the sialic moiety. Two different binding modes were observed for compounds **1** and **2** correlated to their conformational preferences. Compound **1** (Neu5Ac-α-(2,3)-Gal-β-(1–4)-GlcNAc), which adopts an extended conformation, binds HA with its terminal Neu5Ac residue. On the other hand, compound **2** (Neu5Ac-α-(2,6)-Gal-β-(1–4)-GlcNAc) binds the protein in a bent conformation, involving in its interactions not only Neu5Ac, but also Gal and GalNAc residues, and indicating that the nature of the linkage among Neu5Ac and Galactose residues regulates the binding mode.

#### 2.2.2 Interaction Studies in the Presence of H1

The interaction between ligand **1** and cells expressing H1 indicates that Neu5Ac residue is the most involved in the interaction also in this case, since a strong interaction is observed for H4 and H7 (the intensity of the absolute STD is about 2% but these two protons are isochronous and we can not isolate their contribution), the acetyl group (2.9%), H9 (0.8%), H6 and H8 (they are overlapped and their contribution is estimated 0.4%).

The analysis of compound **2** in the presence of cells expressing H1 suggests that the epitope (Figure 6 and Figure S6 of Supplementary Materials) comprises the acetyl group and H7 of the Neu5Ac moiety, the anomeric and H2 protons of Gal, and only the acetyl group of GlcNAc. All of the saccharides have protons interacting with the protein, however it is a quite shallow interaction, with low values of absolute STD percentage (ranging from 0.36% for H2 Gal and H7 NeuAc, to 0.68% for acetyl groups). This epitope is compatible with the interactions suggested in the literature for similar compounds (i.e., Protein Data Bank (PDB) structure 2WRG, Liu et al., 2009) and H1 hemagglutinin.

## 3. Discussion

The fight against the spread of the influenza virus requires an effort to understand the molecular mechanism that favors the diffusion of the virus, and we can benefit from this understanding in order to develop new antiviral small molecules. Since vaccines can be compromised by the rapidity with which the influenza virus mutates, the use of antiviral small molecules represents a valuable alternative. The interaction among natural receptors and HAs is a subject of great scientific interest [9,11,25,26,27], since it can lead to the discovery of novel drugs targeting HA. 

In this paper, we analyzed two trisaccharides (**1** and **2**) derived from respective avian and human receptors of influenza virus hemagglutinin. In order to obtain useful information about the structural requirements needed to develop HA antagonists, we studied the conformational features of these two molecules, followed by their binding properties to H5 (responsible for bird flu) and H1 (responsible for human flu) subtypes of HA. The two trisaccharides differ in the linkage position of the Neu5Ac and Gal residues, which is 2–3 for compound **1**, and 2–6 for compound **2**. It is known that the position of the linkage among sugars influences the conformational features of glycans [27,28], and that on average, α2–3 and α2–6 sialylated glycans show cone-like and umbrella-like topologies respectively. However, small molecules can fluctuate among a limited number of different conformations, which cannot be represented by a single rigid structure. Here, for the first time, we applied a new method to glycans in order to generate a set of conformations whose ensemble is compatible with the experimental NOE data. This method [12] makes use of NOE intensities, measured in triplicate, and of an iterative molecular dynamics scheme to generate a set of conformations by optimizing force field parameters. This procedure is very suitable in the case of flexible molecules, since the standard approach of transforming NOE intensities into spatial restraints, and building conformational models to minimize these restraints greatly neglects the richness of molecular conformations.

From our calculations, we obtained an ensemble of bent conformations markedly populated at equilibrium for compound **2**, and an ensemble of elongated conformations for compound **1**. These results also confirmed the tendency of the trisaccharide to form bent conformations observed for long α2–6 sialylated glycans [28,29] and to maintain an elongated (so-called cone-like) conformation for α2–3 sialylated glycans, and also confirmed the robustness and reliability of our method in its application to glycans and glycomimetics.

The conformational properties and flexibility of ligands can be linked to their identification and engagement with proteins, permitting the optimization of the structural and chemical complementarity of target complexes. The different conformations obtained for these two molecules are due to the chemical nature of the α2–3 and α2–6 bonds, and can be correlated to the binding features observed by STD-NMR for the two trisaccharides in the presence of H5 and H1 subtypes of hemagglutinin.

STD-NMR was used to study compounds **1** and **2** in the presence of 293T cells transfected with H5 and H1 used directly in suspensions, without the need for isolating the protein receptor. STD experiments permitted the identification of the epitope, pinpointing the interactions between parts of each ligand and the receptor. We have already demonstrated that the HAs transfected on the membrane of HEK-293T cells retain their native conformations [11]) and binding properties, evidenced by the rosettes formed with chicken erythrocytes. STD experiments with HA proteins expressed on transfected cells have several advantages over the use of purified proteins and VLPs, obtaining at the same time noiseless experiments, and results under physiological conformation.

We showed that the two molecules are able to bind HAs on transfected cells with different binding modes. In the case of molecule **1**, which has an elongated conformation and is not able to fold, essentially the epitope involves the NeuAc residue in the presence of both H1 and H5, with a similar binding pattern.

By contrast, compound **2**, being an α2–6 sialyl derivative, is characterized by a bent conformation. This conformational preference allows it to interact with both proteins that have an epitope involving the sialyl moiety, and the GlcNAc and Gal residues (as evidenced by STD epitope). Compound **2** interacts with H5 and H1 in a similar manner, but in the presence of H5 the sialic residue is extensively involved.

Our data are in agreement with the binding mode found in co-crystal structures of α2–3 and α2–6 sialylated glycans proposed by [30], supporting the validity of our method of studying the interaction among glycans or glycomimetic and Hemagglutinins at the atomic level. As a perspective, these data will be useful for designing glycomimetics that can reproduce the binding mode of natural glycans with a high affinity for HAs, in order to obtain molecules able to disrupt the carbohydrate–hemagglutinin interactions.

## 4. Materials and Methods

Compounds Neu5Ac-α-(2,6)-Gal-β-(1–4)-GlcNAc and Neu5Ac-α-(2,3)-Gal-β-(1–4)-GlcNAc were purchased from Carbosynth Ltd, Berkshire (UK).

### 4.1. Construction of Stable Hemagglutinin-Expressing Transfectant Cell Lines

Constructs pcDNA3.1-H5 plasmid, carrying the hemagglutinin genes from influenza viruses A/*Cygnus olor*/Italy/724/2005_H5N1 and A/California/7/2009_H1N1, were obtained by subcloning the genes [31] into the multiple cloning site (MCS) of the commercial plasmid pcDNA3.1(+) (Invitrogen, Carlsbad, CA, USA), in which gene expression is directed by the cytomegalovirus (CMV) promoter. The human cell line HEK-293T (ATCC CRL-3216) was transfected with plasmid DNA (10 μg/mL). Transfectants were selected in G418-containing medium and passaged 3 times in the same medium. HA-transfectants showed unaffected growth rate and mortality in comparison to untransfected HEK-293T. Cells expressing HA on the cell surface were sorted (by FACS) with anti-HA stem monoclonal antibody CR6261 [32] (Acrobiosystems, Newark, DE, USA), specific for the native conformation, and expanded as the stable transfectant cell lines H5-293T and H1-293T. Polymerase Chain Reaction (PCR, with serotype-specific primers), Western blot (in reducing conditions, with chicken anti-HA antisera), flow cytometry (with CR6261), immunoprecipitation with CR6261 (followed by Western blot analysis in non-reducing conditions) were carried out by standard methods. For the hemagglutination rosette assay with chicken erythrocytes (ChRBC), H5-293T, H1-293T, or untransfected 293T cells were mixed with ChRBC, spun into a pellet, resuspended, and spun through a Histopaque cushion (293T cells float on Histopaque-1.077, while ChRBC form a pellet). Histopaque-1077 (Sigma-Aldrich-Merck KGaA, Darmstadt, Germany) is a sterile, endotoxin tested solution of polysucrose and sodium diatrizoate, adjusted to a density of 1.077g/mL. HA-dependent 293T rosettes were recovered from the red pellet.

Immunoprecipitation with CR6261 of membrane proteins solubilized by Triton X-100 extraction, followed by Western blot analysis in non-reducing conditions, demonstrates that the surface proteins are correctly conformed as trimers, and are then dissociated into dimers and monomers during electrophoresis. Furthermore, the HA molecules are shown to be able to bind sialic acid, because they agglutinate chicken red cells forming rosettes. We repeated a hemagglutination test on cells in buffer, and confirmed that HAs maintain their conformation after 15 h (which is the NMR condition and experiments time) [11].

### 4.2. NMR Experiments

All NMR spectra were registered on Bruker Avance III 400 MHz and Avance 600 MHz using 7–9 mM solutions of ligands in deuterated phosphate buffer (pH 7.4). The assignment was performed through one- and two-dimensional ^1^H and ^13^C NMR spectra by the standard manual method [33,34]. The complete assignment of the molecules and the list of NOEs are reported in Tables S1 and S2 of Supplementary Materials. For the conformational analysis, three independent NOESY spectra (with 32 scans and 256 increments) were collected using a mixing time of 700 ms and the intensities of cross peaks were measured. To avoid the presence of spin diffusion, NOESY spectra with 100, 200, and 400 ms were also recorded (the NOE build up curves for the NOE between H3axNeu5Ac and H3eqNeu5Ac are reported in Figure S1 for both compounds). The intensities of the cross peaks were calculated and plotted as a function of mixing time, verifying that for these molecules, the intensities at 700 ms are in the linear region of the curve, and can be used for calculation. 

Compounds **1** and **2** were analyzed in the presence of cells’ suspension, and their proton resonances did not show significant shifts relative to the free forms. The STD–NMR spectra were acquired in the presence of about 10 × 10^6^ T293 untransfected and transfected cells in a total volume of 200 mL, using the Watergate sequence for water suppression, and varying the saturation times from 0.98 to 2.94 s. The on-resonance irradiation of the protein was performed at a chemical shift of −0.05 ppm. Off-resonance irradiation was applied at 200 ppm, where no protein signals were visible. Negative controls were performed to avoid the presence of signals in the blank or artifacts. STD spectra of the free ligands and of the cells in the absence of ligands did not show any signals. The velocity of sedimentation of the cells in the NMR tube was not studied, but the cells were maintained in suspension thanks to the rotation into the NMR probe, the spin rate being 20 revolutions/second = 20 Hz.

The tr-NOESY spectra were acquired with 200 ms mixing time. To obtain a tr- NOESY we used 20 × 10^6^ cells, however, unfortunately, we obtained noisy spectra, which did not permit us to perform the integration of the peaks and the analysis of the bound conformation. In any case, the qualitative analysis of NOE cross peaks suggested that there is no difference between free and bound conformations. ^1^H and NOESY experiments were performed using an excitation sculpting sequence for solvent suppression.

### 4.3. Conformational Analysis

The molecular dynamic simulations are performed with Gromacs 4.6.5 [35]. The interactions of the molecule are modelled with the GAFF force field [12,36] in explicit water. After energy minimization and water thermalization, we performed 50 ns of simulation at 300 K, recording 1000 conformations to be analyzed.

The conformational ensembles of the molecules are built on the basis of the NOE intensities with the strategy described in [12], treating independently the α and β isoforms of the two compounds. An initial model for each of the four molecules was built within the GAFF force field [36]. Iterative MD simulations are performed with the Gromacs 4.5 package in implicit solvent, updating the torsional terms of the potential (except those affecting the amidic bonds) to minimize the χ^2^ between the experimental and the back-calculated NOE intensities (cf. also Figure S8 in the Supplementary Materials). To avoid overfitting, the iterations are stopped when χ^2^ = 1, that is, when the back-calculated NOE intensities are within the error bars of the data, obtained from the triplicate experiment.

The NOEs obtained from three independent experiments, together with the associated mean and standard deviations, are reported in Figure S3 in the Supplementary Materials The forward model to back-calculate the NOE intensities for each pair of hydrogen atoms at distance *d* from the ensemble of simulated conformations is:

(1) $$I=I_{0}\langle \frac{1}{d^{6}}\rangle$$

where the angular bracket indicates the average over the simulated conformations, and *I*_0_ is 18.2 for compound **1,** and 3.58 for compound **2**. This forward model holds [37], as the inter-atomic distance correlation time is slower than the rotational correlation time (cf. Figure S9).

## Supplementary Materials

Supplementary materials can be found at http://www.mdpi.com/1422-0067/19/5/1267/s1.

## Abbreviations

NMR	Nuclear Magnetic Resonance
STD	Saturation Transfer Difference
NOE	Nuclear Overhauser Effect
IAV	Influenza A virus
HA	Haemagglutinin
NA	Neuroaminidase
