# Supplementary material for: A Combined NMR-Computational Study of the Interaction between Influenza Virus Hemagglutinin and Sialic Derivatives from Human and Avian Receptors on the Surface of Transfected Cells
Source: Int J Mol Sci. 2018 Apr 24;19(5):1267. doi: 10.3390/ijms19051267 (PMC5983646; doi:10.3390/ijms19051267)
Supplement: Supplementary file 1 [file ijms-19-01267-s001.pdf]

Supplementary Materials

Table S1. NMR assignment of Neu5Ac-α-(2,3)-Gal-β-(1-4)-GlcNAc (compound **1**) in deuterated phosphate buffer (pH=7.4).

		^{1}H (ppm)	^{13}C (ppm)
NeuAc	3	2.67 (eq), 1.72 (ax)	39.3
	4	3.55	67.7
	5	3.75	51.3
	6	3.65	74.7
	7	3.55	62.8
	8	3.65	60.8
	9	3.77, 3.58	62.2
	CH_3	1.95	21.8
Gal	1	4.47	102.4
	2	3.48	68.9
	3	4.04	75.3
	4	3.90	69.6
	5	3.84	71.3
	6	3.79, 3.75	60.1
GlcNAc (α)	1	5.12	90.2
	2	3.81	53.5
	3	3.66	78.0
	4	3.68	67.6
	5	3.60	72.3
	6	3.86	59.5
	CH_3	1.95	21.8
GlcNAc (β)	1	4.63	95.4
	2	3.50	67.2
	3	3.63	67.8
	4	3.68	67.6
	5	3.60	72.3
	6	3.90	59.5
	CH_3	1.95	21.8

Table S2. NMR assignment of Neu5Ac-α-(2,6)-Gal-β-(1-4)-GlcNAc (compound **2**) in deuterated phosphate buffer (pH=7.4).

		^{1}H (ppm)	^{13}C (ppm)
NeuAc	3	2.58 (eq), 1.63 (ax)	39.4
	4	3.56	67.5
	5	3.74	54.9
	6	3.62	71.6
	7	3.48	67.6
	8	3.89	69.0
	9	3.79, 3.58	61.9
	CH_3	1.94	21.8
Gal	1	4.36	102.6
	2	3.46	70.0
	3	3.6	71.8
	4	3.84	67.9
	5	3.74	72.9
	6	3.91, 3.46	62.8
GlcNAc (α)	1	5.12	89.7
	2	3.85	52.6
	3	3.81	70.9
	4	3.58	80.2
	5	3.54	73.9
	6	3.79	59.3
	CH_3	1.98	22.0
GlcNAc (β)	1	4.66	94.0
	2	3.64	55.3
	3	3.81	70.9
	4	3.58	80.2
	5	3.54	73.9
	6	3.83, 3.73	59.6
	CH_3	1.98	22.0

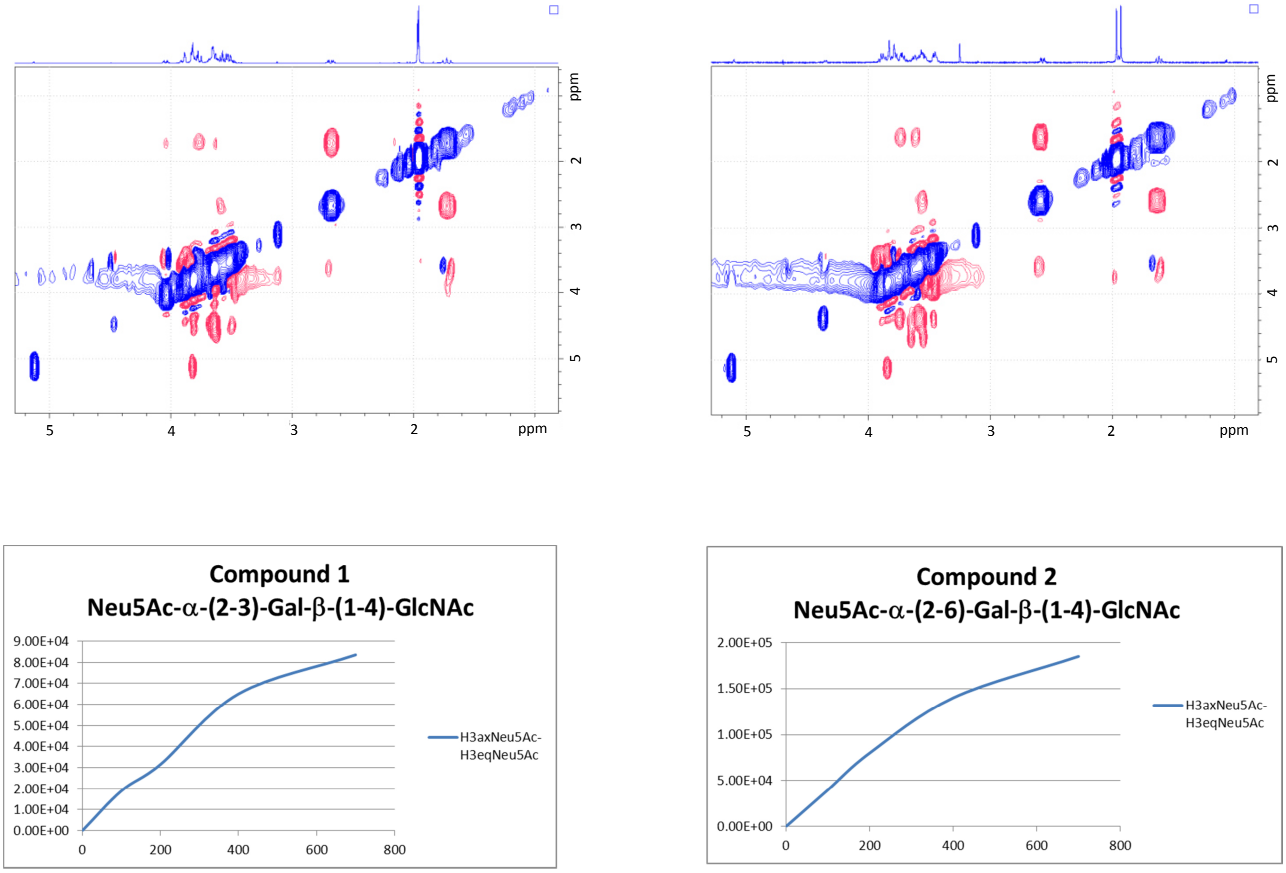

Figure S1: The NOESY spectra of compound **1** (up, left panel) and compound **2** (up, right panel) at mixing time = 700 ms. Build up curves obtained for H3axNeu5Ac-H3eqNeu5Ac for compound **1** (bottom, left panel) and **2** (bottom, right panel) respectively.

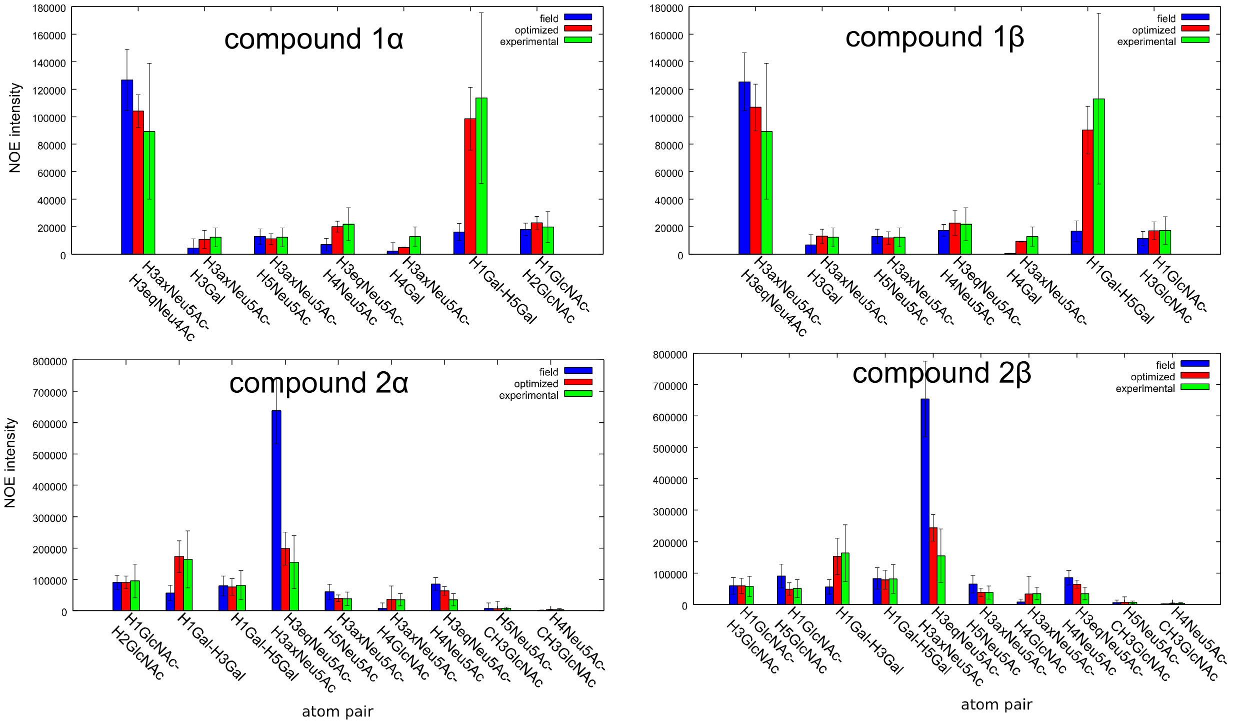

Figure S2: The experimental NOE intensities (green bars) are compared with those calculated from the initial MD simulation performed with the standard GAFF force field, without applying the iterative correction (blue bars; the associated χ^2 being 3.4, 4.1, 3.2 and 3.0, respectively) and those obtained after the optimization algorithm (red bars).

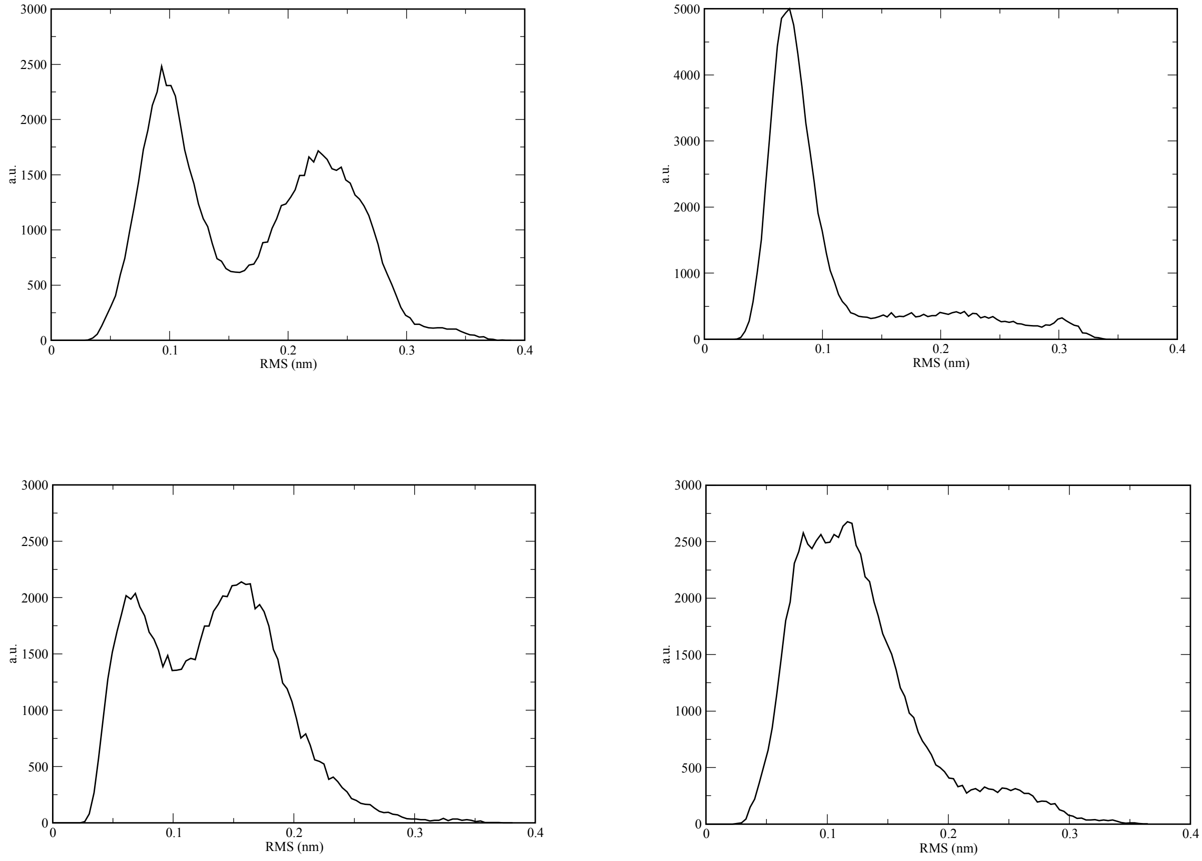

Figure S3: The distributions of root mean square deviations (RMS) between all the pairs of conformations sampled by compound **1** (top panels) and compound **2** (bottom panels). Left panels refer to anomer α while right panels to anomer β.

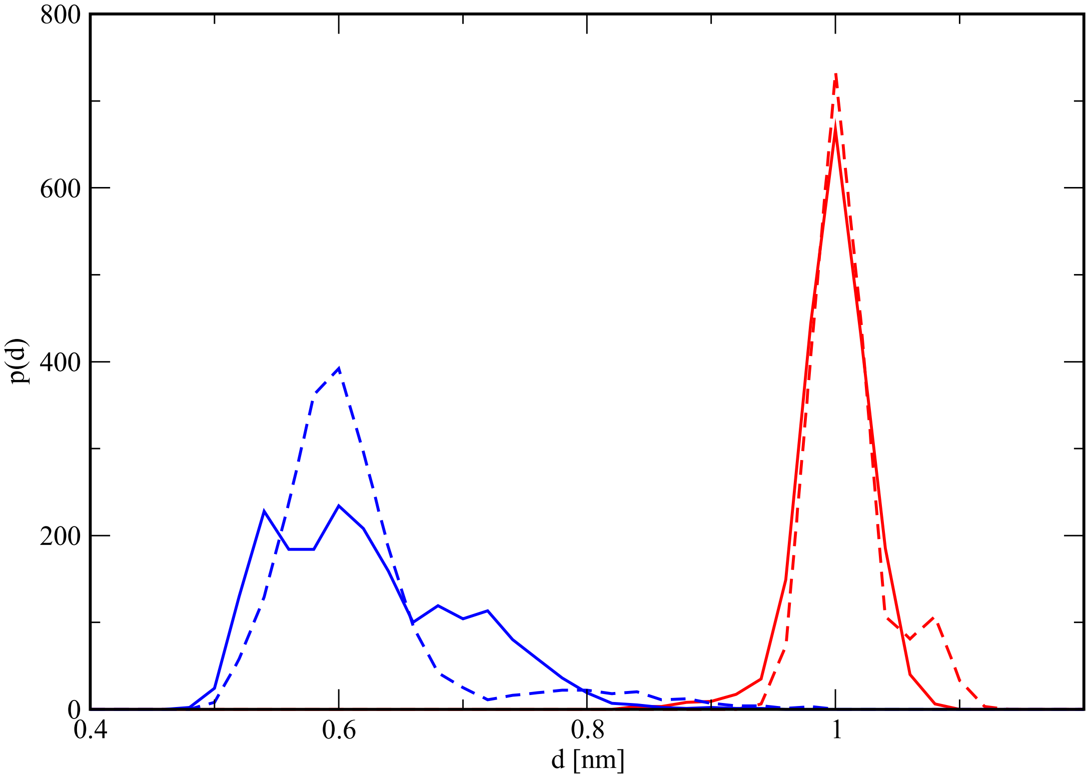

Figure S4: The histogram of the distances between Neu5Ac and GlcNAc in compound **1** (red lines) and compound **2** (blue lines), calculated from anomer α (solid lines) and anomer β (dashed lines).

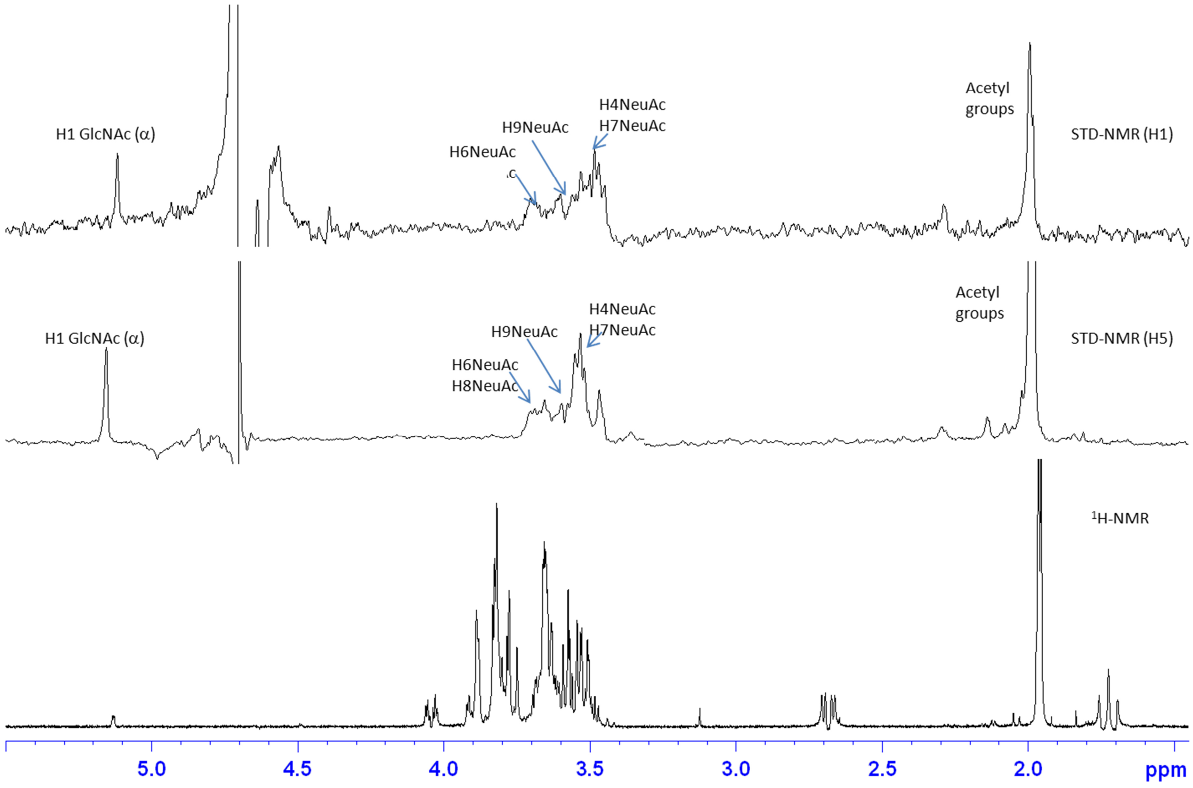

Figure S5. 1H-NMR spectrum of compound **1** (bottom). STD spectrum of compound **1** in presence of cells expressing H5 (middle) and H1 (up).

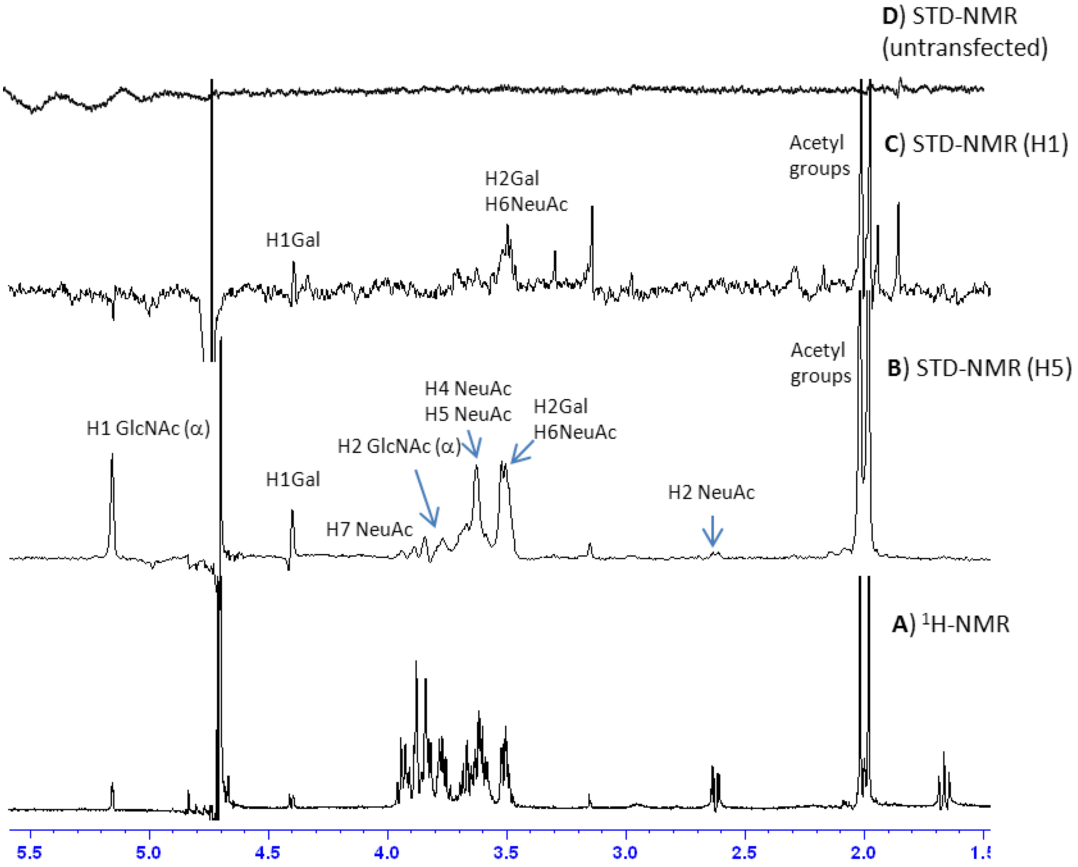

Figure S6. 1H-NMR spectrum of compound **2** (**A**). STD spectrum of compound **2** in presence of cells expressing H5 (**B**), H1 (**C**) and in presence of untransfected (control) cells (**D**).

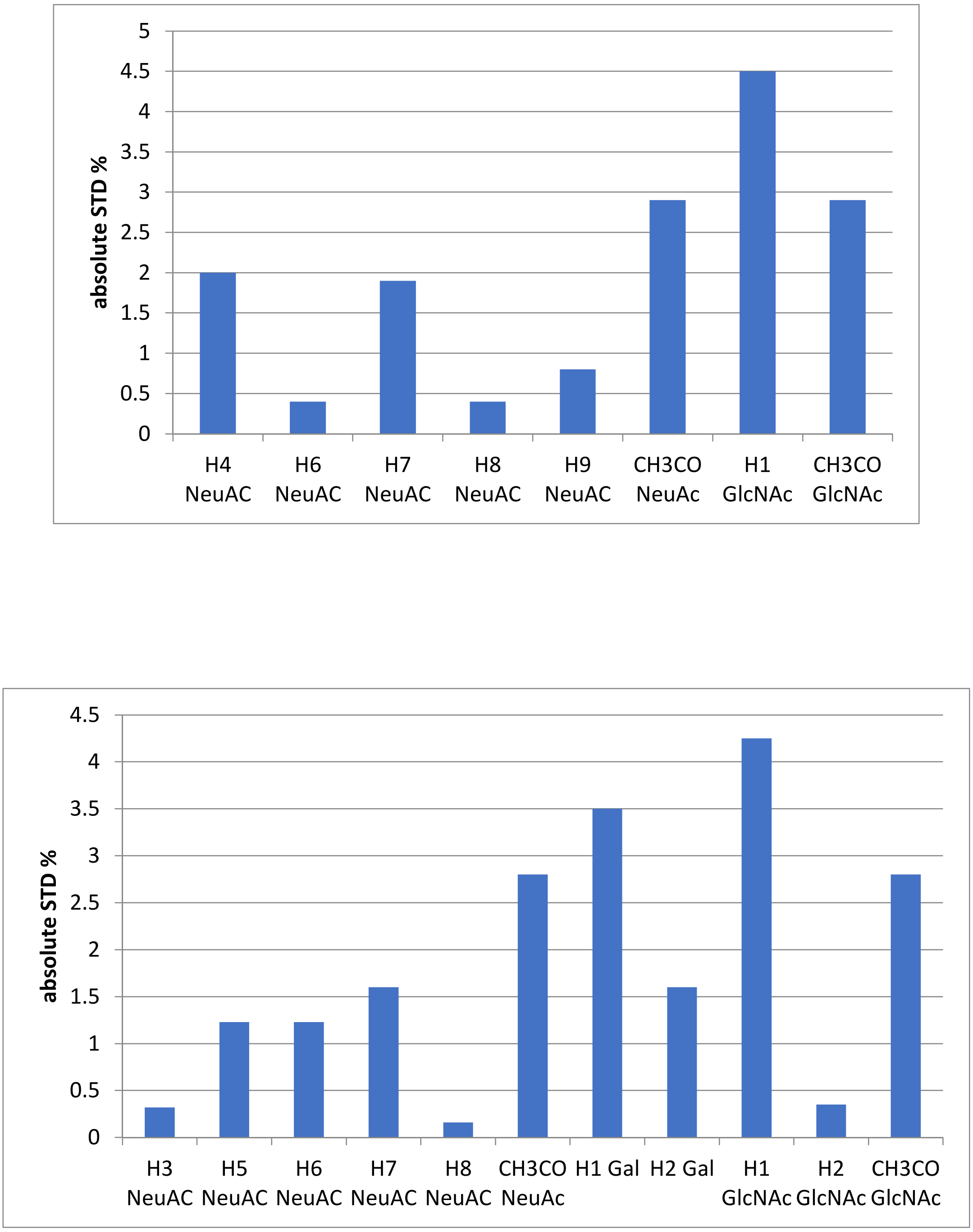

Figure S7: STD intensities of compound **1** (upper panel) and compound **2** (lower panel) in presence of H5.

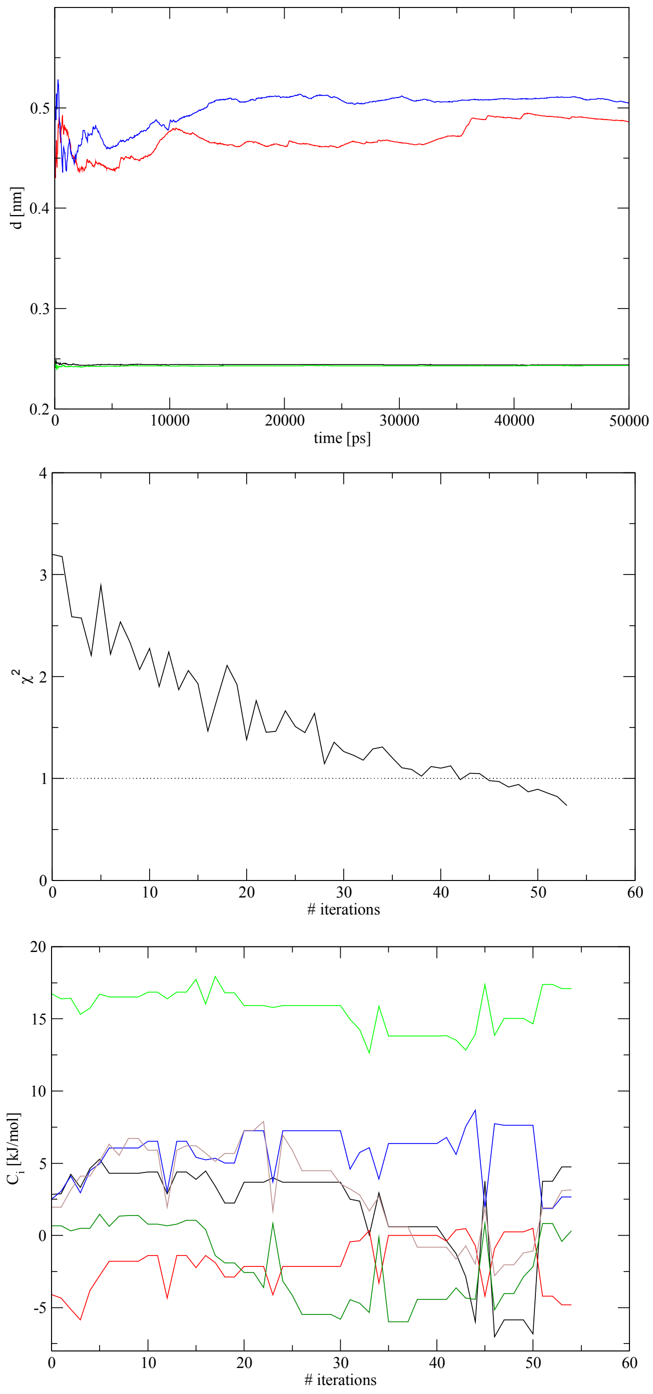

Figure S8: **(upper panel)** The average of interatomic distances between a short-ranged pair (atoms 76-87, black curve) and a long-raged pair (atoms 23-77, red curve) up to a given time, plotted as a function of this time, in the initial simulations performed with the GAFF potential for compound 2α. In green and blue, respectively, the same quantities calculated in the last iteration. (**middle panel**) The decrease of the χ^2 between the experimental and the back-calculated NOE intensities as a function of the number of iterations. The dotted line marks the value χ^2=1, corresponding to the ideal case in which the difference between experimental and calculated intensities match the experimental error bars. (**lower panel**) The change of some energy coefficient of the Ryckaert-Bellemans torsional potential, chosen at random, as a function of the number of iterations.

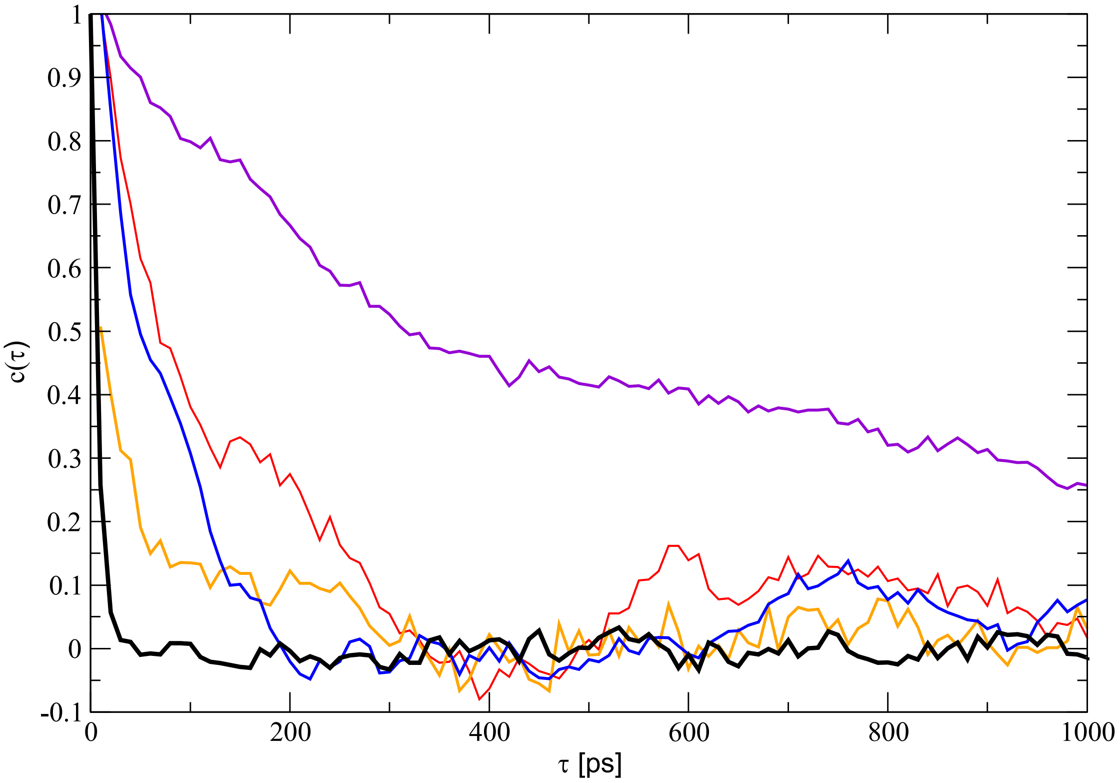

Figure S9: In black, the rotational autocorrelation function of compound 2α, giving an autocorrelation time of $\tau_{rot}\approx10$ ps. The other curves show the autocorrelation function for interatomic distances for two pairs of atoms in the initial model controlled by the GAFF force field (atoms 76-87, blue curve, and atoms 23-77, purple curve) and with the final model (red and orange curves, respectively). Their autocorrelation times range between 40 and 800 ps.
